# Scaling-up Child and Youth Mental Health Services: Assessing Coverage of a County-Wide Prevention and Early Intervention Initiative During One Fiscal Year

**DOI:** 10.1007/s10488-022-01220-3

**Published:** 2022-10-26

**Authors:** Cole Hooley, Deborah Salvo, Derek S. Brown, Lauren Brookman-Frazee, Anna S. Lau, Ross C. Brownson, Patrick J. Fowler, Debbie Innes-Gomberg, Enola K. Proctor

**Affiliations:** 1 Brigham Young University, 84602 Provo, UT USA; 2 Brown School, Washington University in St. Louis, 1 Brookings Drive, 63130 St. Louis, MO USA; 3 Department of Psychiatry, University of California San Diego, 9500 Gilman Drive #0812, 92093 La Jolla, CA USA; 4 UCLA Department of Psychology, 502 Portola Plaza, 90095 Los Angeles, CA USA; 5 Prevention Research Center, Brown School, Department of Surgery, Division of Public Health Sciences, and Alvin J. Siteman Cancer Center, Washington University in St. Louis, Washington University School of Medicine, Washington University in St. Louis CDC U48DP006395, the Foundation for Barnes-Jewish Hospital, 1 Brookings Drive, 63130 St. Louis, MO USA; 6 Los Angeles County Department of Mental Health, 510 S. Vermont Avenue, 17th Floor, 90020 Los Angeles, CA USA; 7 Department of Kinesiology and Health Education, The University of Texas at Austin, Bellmont Hall 822J, 2109 San Jacinto Blvd, Stp D3700, 78712 Austin, TX United States

**Keywords:** scale-up, mental health, mental health services, children and youth, evidence-based practice, implementation science

## Abstract

**Purpose:**

In the U.S., the percentage of youth in need of evidence-based mental health practices (EBPs) who receive them (i.e., coverage rate) is low. We know little about what influences coverage rates. In 2010, the Los Angeles County Department of Mental Health (LACDMH) launched a reimbursement-driven implementation of multiple EBPs in youth mental health care. This study examines two questions: (1) What was the coverage rate of EBPs delivered three years following initial implementation? (2) What factors are associated with the coverage rates?

**Methods:**

To assess coverage rates of publicly insured youth, we used LACDMH administrative claims data from July 1, 2013 to June 30, 2014 and estimates of the size of the targeted eligible youth population from the 2014 American Community Survey (ACS). The unit of analysis was clinic service areas (n = 254). We used Geographic Information Systems and an OLS regression to assess community and clinic characteristics related to coverage.

**Results:**

The county coverage rate was estimated at 17%, much higher than national estimates. The proportion of ethnic minorities, individuals who are foreign-born, adults with a college degree within a geographic area were negatively associated with clinic service area coverage rates. Having more therapists who speak a language other than English, providing care outside of clinics, and higher proportion of households without a car were associated with higher coverage rates.

**Conclusion:**

Heterogeneity in municipal mental health record type and availability makes it difficult to compare the LACDMH coverage rate with other efforts. However, the LACDMH initiative has higher coverage than published national rates. Having bilingual therapists and providing services outside the clinic was associated with higher coverage. Even with higher coverage, inequities persisted.

**Supplementary Information:**

The online version contains supplementary material available at 10.1007/s10488-022-01220-3.

## Background

### The Need to Scale-up Mental Health Services for Youth

Mental illness in children and adolescents (referred collectively to as youth throughout this paper) is prevalent and burdensome (Baranne & Falissard, 2018; Office of the Surgeon General, 2021). Fortunately, there are various evidence-based practices (EBPs) to effectively treat mental illnesses in youth (Chorpita et al., 2011). Some argue that treatment-as-usual yields unreliable outcomes (Bear et al., 2019) and that EBPs perform better (Lang et al., 2021). Unfortunately in the U.S., most youth in need of mental health services do not receive any type of treatment (57%), and even fewer receive an EBP (1 to 3%) (Bruns et al., 2016; Substance Abuse and Mental Health Services Administration, 2020). Further, ethnic minority youth with need are less likely to receive mental health services (Marrast et al., 2016).

Municipalities use various strategies to scale-up evidence-based practices for youth mental illnesses and report facing challenges. Scale-up refers “to intentional efforts to maximize the positive impact of mental health interventions successfully tested in experimental studies to benefit mental health care at the national level or at a regional level within a country and to foster evidence-based mental health policy and program development on a lasting basis” (National Institute of Mental Health, 2016, p. 1). Nearly all of the states surveyed (94%) report engaging in some effort to promote EBPs in their service systems (Cooper & Aratani, 2009). About one quarter of systems report scale-up as one of their top challenges, noting disconnects between system administrators and frontline providers and advocates (Cooper & Aratani, 2009, 2015).

There is a pressing need to understand what influences scale-up (Singla et al., 2018). In the U.S., capturing the extent to which a target population has received mental health services has largely been limited to services provided in the Veterans Administration (VA) (Mohr et al., 2018). The VA service system uses a universal electronic health record (EHR) and has access to military service records, which can be combined to assess the veteran population-level effects of EBP scale-up initiatives. Conducting the same analysis in public, civilian mental health service systems without universal records is much more challenging. The present exploratory study sought to contribute to the scale-up literature by assessing the extent to which youth EBPs have been scaled-up within a public, civilian mental health system, and the factors associated with that scale-up.

### Frameworks

Two frameworks guided this study: the Health Services Coverage Framework and the ExpandNet framework (Tanahashi, 1978; World Health Organization, 2010). The Health Services Coverage framework provides guidance on how to operationalize our outcome variable and the ExpandNet framework helped in the selection of scale-up determinants.

The success of a scale-up effort may be indexed as the coverage of a given intervention. In the Health Services Coverage framework, Tanahashi (1978) defines contact coverage as the ratio of those who have received the service and the target population (Tanahashi, 1978). Coverage rates also involve a specific time interval. For the purposes of our study, the service was child psychotherapy EBPs provided through the Los Angeles County Department of Mental Health (LACDMH) clinics and affiliates. Our target population was eligible youth in Los Angeles County for whom the psychotherapy services are intended. We selected contact coverage as our outcome given its explicit focus on using a community-level population denominator (Tanahashi, 1978).

The ExpandNet framework identifies broad categories of determinants that influence the scale-up of an intervention. Those categories include characteristics of the innovation, the scale-up support team, the user organizations, the external environment, and the multiple facets of the selected scale-up strategy. In this study, the innovations are mental EBPs, the scale-up support team is the Los Angeles County Department of Mental Health (LACDMH), the user organizations are the mental health clinics, the external environment includes the policy, community, fiscal, and cultural factors in LA county, and the scale-up strategy includes the package of strategies the LACDMH has used to date. The determinants domains of interest for our study are the user organizations and the environment. We then used the literature to operationalize variables within these domains.

### LA County Prevention and Early Intervention Initiative

The Los Angeles County Department of Mental Health (LACDMH) is the largest county mental health provider in the United States (Lau & Brookman-Frazee, 2015). Every year, LACDMH provides services to approximately 250,000 residents (Los Angeles County Department of Mental Health, 2022). These services are provided through a combination of county-operated clinics and contracts with other provider agencies and individuals.

In 2010, the LACDMH began an ambitious initiative to scale up the coverage of EBPs across directly operated and contracted mental health programs (Regan et al., 2017), including for children, adolescents, and transition-age youth. This initiative was funded by the California Mental Health Services Act Prevention and Early Intervention (PEI) program that focuses on providing mental health services to individuals who are showing the first signs of a mental illness (Regan et al., 2017). During the first five years of PEI, 87,000 unique children received services (Brookman-Frazee et al., 2016). Previous LACDMH scale-up research has explored the sustainment of the six supported EBPs within the service-provider-system (Brookman-Frazee et al., 2016, 2018).

A critical component of the EBP scale-up strategy for the LACDMH PEI program was the provision of reimbursement only for delivery of an identified set of approved intervention models. LACDMH approved 52 practices which included evidence-based practices, promising practices, and community-defined practices for young children, children, and transition-aged youth (Regan et al., 2017). LACDMH provided implementation guidelines associated with each individual EBP and PEI program eligibility.

This exploratory study is the first (known to the authors) U.S.-based study to determine the coverage of a suite of EBPs in a civilian, public mental health system. The present exploratory study seeks to contribute to the scale-up literature by answering the following questions:

1. What is the PEI coverage rate three years after initial implementation (fiscal year 2013–2014)?
2. What community-level and clinic-level factors are associated with the coverage rate of the targeted population?

## Methods

This study was a cross-sectional, small area geospatial variation analysis. We used road network buffers (service areas) and apportionment to estimate coverage rates for the PEI initiative at the county and clinic service area levels. We then used an ordinary least squares (OLS) regression to identify correlates of clinic service area-level coverage rates. We used geospatial reporting recommendations of the ISLE-ReST reporting guideline (Jia et al., 2020).

### Data Sources

This study combined LACDMH administrative claims data for the PEI initiative and geospatial data from the American Community Survey (ACS) within LA County. Other researchers have combined administrative data with geospatial and other census data to answer mental health services related questions (Guerrero et al., 2013; Walker et al., 2016).

#### Administrative Claims data

We used psychotherapy claims data from the LACDMH for clients ages 0 to 25 who received an approved PEI EBP during the 2013–2014 fiscal year (FY) (Brookman-Frazee et al., 2016). For each encounter, therapists submitted a claim to LACDMH, demonstrating the client received a fitting PEI-approved EBP given the client’s age and presenting problem (Brookman-Frazee et al., 2016). Each claim includes a service code, demographic and diagnostic data about the client, demographic data about the therapist, the code for the EBP, and information about the location where the client received services. As in previous publications, we excluded claims for services like medication management, evaluation and assessment, and case management (Brookman-Frazee et al., 2016). We selected the 2013–2014 fiscal year (July 1, 2013 to June 30, 2014) because it represents a mid-point in the PEI scale-up initiative and is the fiscal year with the highest number of unique youth clients served. The PEI initiative is meant for prevention and early intervention, and as such, it is meant for mental health difficulties that may be responsive to short-term treatment. The approved PEI EBPs have varying treatment durations. Across all EBPs, the median duration is 20 weeks. The recommended treatment duration for the 6 main practices ranges from 10 to 50 weeks. With the exception of one EBP, treatment does not exceed one year (Los Angeles County Department of Mental Health, 2016).

#### Geospatial Data

We used the American Community Survey (ACS) block-group single year 2014-five-year-estimates to provide neighborhood-level demographic data because these estimates are typically more stable. There are 6,425 block groups in LA County (United States Census Bureau, 2018). We used block groups because they were the smallest geographic unit with available census data, and others have used the block group level to characterize neighborhood units, when assessing neighborhood-level predictors of mental health service disparities (Cook et al., 2017).

We used publicly available GIS boundary data of varying geospatial scales to create the geospatial units for analysis (LA County and LA County ACS block groups). We spatially joined those boundary files with the ACS and claims data (by the location of each clinic) using ARCMap 10.6. See supplementary table S1 for a list of the shapefiles we used.

### Measures

For the first research question, we used the county level to provide a system-wide metric of how many people in the target population (youth residents of LA County who would quallfy for a PEI EBP) were served (see supplementary table S2).

We used clinic-service-area (CSA, n = 254) as the unit of analysis to answer the second research question. This geographic unit is important to analyze because it is the point at which clients come into contact with the EBPs. Road-network distance is one method geospatial researchers have used to derive meaningful distances for behavioral health service access (Apparicio et al., 2008; Ngamini Ngui & Vanasse, 2012; Walker et al., 2016). Road-network distance calculates possible routes from a point (e.g., clinics) using the available road system network. See supplementary figure S1 for an example.

We applied service area network analysis to create the buffers around each clinic (Ballas et al., 2018). We identified the clinics from the administrative claims data (those clinics who submitted PEI reimbursable services within the 2013–2014 fiscal year). We geocoded the clinics in ARCMap 10.6 and then calculated a 2-mile road-network-based service area buffer around each clinic using LA County census street maps data. The radius distance of road-network-based service area buffers vary in the health services literature (Ngamini Ngui & Vanasse, 2012; Packness et al., 2017). We based the 2-mile radius buffer for this study on research conducted in LA county (Maguire-Jack & Klein, 2015).

We built our dependent and independent variables using apportionment. The apportionment process aggregated the claims data and the census data to the CSA-level (ESRI, 2019). Apportionment is the process of calculating a *spatial weighted average* of an area-based variable using two or more overlapping shapes. The resultant product from the apportionment process was a dataset with observations at the CSA level, where the dependent variable was the CSA coverage rate, and the independent variables were the agency/provider and census variables aggregated to the CSA. The dependent and independent variables are described in greater detail in the following two sections.

#### Dependent variable: Coverage Rate

Our dependent variable is a coverage rate. The coverage rate is the proportion of the target population who received PEI services during the 2013–2014 fiscal year. We followed guidance from the Health Services Research Group to operationalize our coverage rates (dependent variable) (Health Services Research Group, 1992). There are four sources of information needed to construct the coverage rate. One must know the number of individuals who received the service (numerator), the number of the service’s target population (denominator), the time interval of interest, and the geography on which these numbers are based to determine the coverage of an intervention.

The numerator is the number of individuals who received the service during the specified time frame (Health Services Research Group, 1992). We calculated the numerator for the county and CSA levels by identifying the number of unique youths in the claims data who received one of the approved EBPs during FY 2013–2014 within those geographic levels. Like other studies (Brookman-Frazee et al., 2016), we counted a child as having received services if they had at least one psychotherapy session claimed for PEI reimbursement for an approved EBP. This represents an estimate of the size of the population who had exposure to PEI EBPs.

Denominators can be calculated in several different ways (De Silva et al., 2014). The bluntest method might be to multiply the geographic unit’s total population of youth by the epidemiologic prevalence rates for any mental illness (De Silva et al., 2014). The population data for this type of calculation would come from the ACS and the prevalence rates would come from the mental health literature (Kessler, Avenevoli, Costello, Georgiades, et al., 2012; Merikangas et al., 2010). Other researchers, however, have advocated the importance of modifying the denominator so it more closely reflects the realities of eligibility for service utilization (De Silva et al., 2014; Department of Health, 2012; Green, 1996; Humensky et al., 2013).

In line with those recommendations, we calculated the denominator following a five-step process similar to the one used by the *Improving Access to Psychological Therapies* program (Department of Health, 2012) and others (Humensky et al., 2013) (see supplementary table S3).

1. We obtained the youth population estimate for each census block group from the ACS dataset.
2. We multiplied the youth population estimate by the percentage of the population in the census block group who were enrolled in Medi-Cal, California’s state Medicaid program (Research and Analytical Studies Branch, 2011), given that PEI services covered that population.
3. We multiplied the Medi-Cal enrolled youth population by the epidemiologic prevalence rate for mental disorders as an indicator of need for mental health services (Kessler, Avenevoli, Costello, Georgiades, et al., 2012; Merikangas et al., 2009).
4. We multiplied the population of youth with any qualified mental disorder with the prevalence rate for youth without serious emotional disturbance (Kessler, Avenevoli, Costello, Green, et al., 2012) because PEI is a prevention/early intervention initiative targeting youth without severe impairment whose mental health difficulties are likely to respond to short-term treatment (i.e., one year or less of treatment) (Regan et al., 2017).
5. We multiplied the non-severe prevalence population by the percentage of youth likely to seek services which is approximately 50% (Garland et al., 2005; Merikangas et al., 2010). Others have reduced target population calculations by those who are willing to seek services (Department of Health, 2012; Humensky et al., 2013).

The same prevalence percentages will be used to cover the full fiscal year FY 2013–2014 given the stability of prevalence rates (Merikangas, 2018; Sawyer et al., 2018). We generated a denominator for each census block group and used apportionment to calculate the denominator for each CSA.

#### Independent variables

Using our clinic service areas (CSA), we created independent variables that operationalized clinic and neighborhood factors that have been associated with mental health service utilization. We outline the final list of variables and how they were calculated in Table 1 We initially created the variables using the method outlined in the “calculation” column. Then, we aggregated the values using the apportionment procedures to the CSA level. Researchers have tested multivariate models in small area variation analysis (SAVA) studies and used a similar approach to construct their variables (Green, 1996; Kelly & Jones, 1995).

**Table 1:** Description of variables apportioned for OLS regression analysis

DV/IV	Variable	Description	Data source	Calculation of variable prior to apportionment
DV	Coverage	CSA coverage rate proportion	Claims and ACS	Number of distinct clients served by clinic divided by number of target PEI population
IV	Therapist language	Number of therapists who speak a language other than English	Claims	Number of therapists whose primary language was Spanish, Other, or multiple languages.
IV	Service setting	Proportion of claims provided in a setting outside the clinic	Claims	Number of claims executed outside of the office divided by the clinic’s total number of claims with a known location
IV	Ethnic minority	Proportion of population who identify as an ethnic minority	ACS	Number of individuals who identify as an ethnic minority divided by number of individuls
IV	Born outside US	Proportion of population who were born outside the U.S.	ACS	Number of individuals born outside the U.S. divided by the number of individuals
IV	Impoverished	Proportion of the population below the poverty line	ACS	Number of individuals below the poverty line divided by the number of individuals
IV	Transportation	Proportion of households without access to a vehicle	ACS	Number of households without a vehicle divided by the number of households
IV	Education	Proportion of adults (≥ 25) with at least a college degree	ACS	Number of adults (≥ 25) divided with at least a college degree by the number of adults (≥ 25)
IV	English	Proportion of households designated as having limited English	ACS	Number of households designated as limited-English speaking divided by the number of households
IV	Density	Population density of 0 to 24y/o	ACS	Divide the number of individuals between 0 and 24 by the area

ACS = American Community Survey 2014 5 year-estimates

Claims = Los Angeles County Department of Mental Health reimbursement claims

Our selection of independent variables is based on factors shown to be relevant for understanding coverage rates, per the previous literature. Service system independent variables from the claims data included service setting (proportion of sessions held in locations outside the clinic) and provider language (number of therapists who speak a language in addition to English) (Green et al., 2013; Lyon et al., 2013; Swick & Powers, 2018). Community characteristics that served as independent variables from the census data included residents’ race/ethnicity (proportion non-Hispanic white), nativity (proportion foreign-born), socioeconomic status (proportion below poverty line), access to transportation (proportion of households without a vehicle), education level (proportion of adults with at least a college degree), and English proficiency (proportion of households designated as limited-English speaking) (e.g., Alegria et al., 2016; Cauce et al., 2002; Chow et al., 2003; Cook et al., 2017; Dahal et al., 2018; Derr, 2016; Fleury et al., 2014; Garland et al., 2005; Kirby & Kaneda, 2005; Lyon et al., 2013; Merikangas et al., 2011; Ohtani et al., 2015; Reiss, 2013; Stein et al., 2014).

We included youth population density as a control variable in our model. The population density variable is the number of 0 to 24-year-olds within the CSA, per square mile.

### Analysis

We conducted our analysis at two different levels. The first research question was examined at the county level. The analysis for the second research question was conducted at the clinic service area level. The following section describes the analysis steps for each research question.

#### Coverage Rates

We used descriptive statistics to explore the coverage rate at the county level and multivariate OLS regression to answer the second research question (Kutner et al., 2004). We calculated the coverage score by dividing the number of youth who received services by the target population. We calculated a coverage score at the county level and then for each CSA.

#### Factors Associated with Coverage Rates

Other small area variation analysis studies have used multivariate regression models with varying sample sizes, some with samples as small as 10 (Kelly & Jones, 1995; McLaughlin, 1988; Wennberg & Gittelsohn, 1973). The dependent variable for the analysis was the contact coverage rate for each CSA. While there is empirical guidance on the selection of predictors and correlates in the mental health service access literature, these predictors have not been analyzed in relation to contact coverage. We have used that literature to inform this exploratory study. We used all qualified claims at the CSA-level for clinics whose service buffer fell completely within the boundaries of LA county. We also included claims for the small percentage of clients (4.7%) who received services from more than one clinic because the coverage rate for the CSA geographic level assumes the perspective of the clinic and removing the clients who received services from more than one clinic would not accurately represent the clinics’ coverage rates. Initially, there were 261 CSAs in the dataset. One of the CSAs was outside LA county, and six additional CSAs had buffers that extended beyond the county line. The census block group data was specific to LA county, so those buffers that extended beyond LA county lacked estimates for the census blocks outside the county. The predictors and the coverage denominators for those buffers would be underreporting the characteristics of the buffer, as such, we dropped those six CSAs from the analysis. The initial analytic sample for the first research question was n = 254 CSAs.

We assessed OLS assumptions for the model and made necessary corrections. We tested the assumptions using the Shapiro-Wilks test, Cook’s D test, Breusch-Pagan/Cook-Weisberg test, visual inspection of linearity, and correlations between predictors (Chen et al., 2003; Kutner et al., 2004). We assumed some degree of spatial autocorrelation given the close geographic proximity and overlap of buffers in certain regions of the county. We used a series of corrections due to departures from OLS assumptions. We used a log transformation to correct for the skewed distribution of the dependent variable. We retained influential observations after determining no data errors (Kutner et al., 2004). There were some CSAs with computed coverage rates above 1.0. It appeared that those CSAs were in geographic regions where it is likely that clients from outside the buffer came to receive services because there were few alternatives.

We dropped the limited English variable from the model given its inflated variation inflation factor and high correlation with the foreign-born variable. We retained the other variables because they reflect important social determinants of mental health service access. We applied a robust variance estimator to minimize the influence of auto correlation in the data and correct for heteroskedasticity (Huber, 1967; Mehmetoglu & Jakobsen, 2017; White, 1980). There were three clinics whose claims were missing the service location, so they were excluded from the analysis. The final analytic sample for the second research question was n = 251. There were no missing data in the final analytic sample. We used Stata 16.1 (Stata Corp., College Station, TX) for all OLS data management and analysis.

## Results

### Characteristics of Children and Youth Served

Overall, 40,132 unique children and transitional age youth received psychotherapy services under PEI during 2013–2014 (see Table 2 for client descriptive statistics). There were more males (55%) than females (45%), most clients identified as Latino/a (71%) followed by African American (15%), and the majority listed English as their primary language (72%) followed by Spanish (27%). Prevalence of admission diagnoses varied. The most prevalent were mood disorders (30%), followed by disruptive behavior disorders (23%), adjustment disorders (12%), anxiety disorders (11%), hyperactive/attention disorders (10%) and trauma (8%). Client average age was 11 years old with a range from 0 to 25 years (SD = 4.68).

**Table 2:** County-level Prevention and Early Intervention initiative client demographic and service statistics for fiscal year 2013–2014 (n = 40,132)

	N(%) / Median(SD)
Gender	
Male	22,506 (54.9)
Female	18,068 (45.0)
Missing	8 (0.02)
Ethnicity	
Latino/a	28,298 (70.5)
African American	6122 (15.3)
White	3056 (7.6)
Not reported	972 (2.4)
Other	761 (1.9)
Asian	669 (1.7)
American Indian	193 (0.5)
Pacific Islander	61 (0.2)
Primary Language	
English	28,898 (72.0)
Spanish	10,666 (26.6)
Other	430 (1.1)
Not reported	138 (0.3)
Admission Diagnosis	
Mood	12,133 (30.2)
Disruptive behavior	9224 (23.0)
Adjustment disorder	4625 (11.5)
Anxiety	4522 (11.3)
Attention/Hyperactive	3946 (9.8)
Trauma	3034 (7.6)
Other	2452 (6.1)
Autism/PPD	185 (0.5)
Substance use	11 (0.03)
Age (years)	11 (4.7)
Number of sessions per child	13 (25.8)
Number of therapists per child	1 (1.8)
Number of clinics per child	1 (0.23)

### PEI EBP Coverage Rates by County, Service Planning Areas, Clinic Service Area

**County-Level.** The county-level analysis assumed the perspective of the LACDMH and the coverage of the selected PEI EBPs over the entire county (n = 1). The numerator of the county coverage rate is the number of unique children served by any of the 261 clinics that received at least one session of PEI-reimbursed EBP psychotherapy in FY 2013–2014. The denominator for the county was 236,312 children and youth aged 0–25 who were estimated to be eligible for PEI services (after the denominator reduction steps). The county coverage rate for FY 2013–2014 was 17.0%. Most youth received multiple EBP sessions. Approximately 7% of youth received one session. The median number of sessions was 13 (mean 20) with 75% of the youth receiving 5 or more sessions. One-quarter of the youth received 26 or more sessions.

**Clinic Service Area.** The coverage rates as well as the values of the community-level predictor variables varied across the CSAs (see Table 3 for descriptive statistics). The average coverage rate for CSAs was 14%. Four CSAs had coverage rates above 100% (max 306%), the CSAs with higher coverage values increased the average. Removing the four CSAs the average coverage rate was 11%. Clinics that had over 100% coverage were likely serving clients who lived outside the 2-mile buffer. For example, there are some areas of the county with few clinics, so clients from outside the buffer would have to travel to them. It is also possible that some clinics are closer to public transportation hubs that would bring in clients from outside the buffer. The median CSA coverage was 6% with or without the four CSAs with coverage rates above 100%.

**Table 3:** Descriptive statistics for the clinic service areas fiscal year 2013–2014 (n = 254^a^)

	Mean	SD	Median	Min	Max
Clinic service are coverage rate	14.0%	30.0	5.8	0.0	305.9^b^
Ethnic minority	76.8%	17.8	78.9	24.2	99.3
Born outside US	34.3%	11.2	32.9	13.0	59.6
Below poverty	19.8%	9.5	17.0	5.0	44.7
No vehicle	11.1%	8.0	8.4	2.1	39.5
College degree	25.6%	14.6	23.5	4.2	66.2
Sessions outside office^a^	35.4%	32.3	28.4	0.0	97.5
Number bilingual therapists	11	11	7	0	74
Population density (per sq mile)	4058.65	2462.44	3390.33	27.58	11480.87

a = Sample size for sessions outside office is n = 251

b = Coverage rate exceeded 100% in four clinics where it was likely that youth from outside the geographic catchment area traveled to the clinic for services

Data sources: American Community Survey, 2014 5-yr estimates. Los Angeles County Department of Mental Health claims data

### Factors Associated with Coverage at the CSA Level

The results of the multivariate regression suggested that the independent variables explain 47.9% of the variance in CSA coverage rates (R^2^ = 0.48, F(8, 242) = 17.41, p < .001) with all predictors being statistically significant at the p < .05 level except for the poverty variable (see Table 4). After controlling for other variables in the model, per unit increase in the proportion of ethnic minorities in a CSA predicted a lower coverage rate (ß=-2.11, p < .05). Controlling for other variables in the model, per unit increase in the proportion of individuals born outside the US predicted a lower coverage rate (ß=-2.05, p < .05). Controlling for other variables, per unit increase in the proportion of households without a vehicle predicted a higher coverage rate (ß=5.55, p < .05). Controlling for other variables, per unit increase in the proportion of individuals with a college degree predicted a lower CSA coverage rate (ß=-3.26, p < .05). Controlling for other variables, per unit increase in the proportion of sessions held outside of clinics (e.g., home, school) predicted a higher coverage rate (ß=0.80, p < .01). Controlling for other variables, per unit increase in the number of therapists who speak a language in addition to English in a clinic predicted a higher coverage rate (ß=0.08, p < .01).

**Table 4:** Regression coefficients of community and clinic predictors on clinic service area coverage rate (log transformed) for Prevention and Early Intervention initiative claims for fiscal year 2013–2014

	Coef.	95% CI	
Ethnic minority	-2.11*	-3.97	− 0.25
Born outside US	-2.05*	-4.02	− 0.08
Below poverty	-4.47	-8.99	0.04
No vehicle	5.55*	0.88	10.21
College degree	-3.26*	-5.89	− 0.64
Sessions outside office	0.80**	0.26	1.35
Bilingual therapists	0.08**	0.06	0.10
Population density (per sq mile)	-0.00*	− 0.0003	− 0.0001
R^2^ = 0.48			
N = 251			

* p < .05

**p < .01

Data sources: American Community Survey, 2014 5-yr estimates. Los Angeles County Department of Mental Health claims data

## Discussion

Child and youth mental illnesses are prevalent, debilitating, and costly (Beecham, 2014; Merikangas et al., 2009; Vos et al., 2012). Fortunately, there are effective interventions to treat these disorders (Chorpita et al., 2011). Systems of mental health care across the United States have engaged in various initiatives to implement these effective treatments (Cooper & Aratani, 2009). The coverage rates of these initiatives in the United States and the identification of any factors associated with their respective degrees of population coverage are largely unknown (Bruns et al., 2016; De Silva et al., 2014). The present study sought to address these gaps in the literature by assessing the scale-up of EBPs for child and youth mental illnesses in LA county through their PEI initiative. An adapted framework based on the ExpandNet and the Health Services Coverage frameworks informed the selection of outcome and predictors (Tanahashi, 1978; World Health Organization, 2010). The LACDMH PEI initiative is a herculean effort to provide needed mental health services to children and youth. The study yielded coverage rates of the PEI initiative at various geographic levels and identified clinic- and community-level factors associated with those scores.

### Coverage Rates of PEI EBPs

This study examined coverage rates at two levels. The first coverage rate is at the county level. The LACDMH funded EBP delivery within psychotherapy services to 40,132 children/youth during FY 2013–2014 with each of these clients receiving a median of 13 sessions. The county-level coverage rate for the six EBPs was approximately 17% of the target population. The second level was at clinic service area (CSA), which is discussed in the next section.

It is difficult to discern how this coverage rate compares to other such initiatives, given the dearth of coverage rate reporting in the mental health services literature (De Silva et al., 2014). One national study found that child/youth mental health EBPs in the US had a coverage rate of 1–3% (Bruns et al., 2016). That study’s denominator was the number of youth identified by the State as having serious emotional disturbance (Bruns et al., 2016). Our coverage rate for PEI used a more refined denominator following methods suggested by others which used reductions like the help-seeking rate (De Silva et al., 2014; Department of Health, 2012; Humensky et al., 2013). The difference between these denominators made the two coverage rates difficult to compare.

Studies conducted in the United Kingdom and Canada offer other proximal coverage comparisons. Pile and colleagues (2020) assessed the coverage of youth depression care in four London boroughs. Approximately 25% of youth ages 12 to 18 received care between April 2014 and April 2015, and 2% of children 0 to 11 received care. They constructed their denominator using national census data and youth depression prevalence rates (Pile et al., 2020). Adult depression and anxiety care in the United Kingdom had an estimated coverage rate of 16% of the target population (Clark, 2018). That denominator used census data and prevalence rates of depression and anxiety in the adult population (Clark, 2018). In Ontario, investigators found that approximately 4% of children with a mental health need received services (Duncan et al., 2020).

US-based efforts in the Veterans Administration to scale-up trauma care has yielded a range of coverage rates. One estimate suggests that of all veterans with a PTSD diagnosis, 3-4% received Cognitive Processing Therapy or Prolonged Exposure (Sayer et al., 2017). Others in the Veterans Administration have found coverage rates of 6% (Shiner et al., 2013; Watts et al., 2014) and 12% (Rosen et al., 2017). When researchers used veterans with a PTSD diagnosis who received psychotherapy as the denominator, instead of veterans with only a PTSD diagnosis, the coverage rate was 14-59% (mean 36%) (Mohr et al., 2018).

Our findings contribute to the literature on coverage rates in a few ways. First, we provide a coverage score for a public mental health system in the U.S.. Most of the mental health service coverage literature in the U.S. is based on Veterans Administration services. Second, our coverage rate offers a more tailored approach to denominator construction that could be useful to other service systems. Being able to quantify the target population is an important step in the scale-up process. Our denominator reflects a more realistic target for the LACDMH. Third, we were able to combine various sources of data that could be useful for service systems that lack a universal health record like the Veterans Administration.

### Clinic Service Area Factors Associated with Coverage

The proportion of ethnic minorities in the community was associated with a lower coverage rate. Racial/ethnic disparities in mental health service access have been a persistent issue within the mental health services literature (Cook et al., 2013; Misra et al., 2021). Stigma, cost, an insufficient supply of culturally responsive clinicians, lack of providers who speak the clients’ language, racial discrimination, and distrust based on historic abuses are among some of the barriers experienced by ethnic minority communities (Misra et al., 2021). This disparity is even more glaring given the prevalence of racism and its negative mental health outcomes (Cave et al., 2020). There is another possible explanation for the relationship between lower coverage and higher density of ethnic minorities. Higher ethnic density in communities has been found to be a protective factor for mental health (Bécares et al., 2018). Living in higher own-group communities can attenuate the impact of racism and discrimination and bolster social capital and its benefits (Baker et al., 2021). It may be that there was lower coverage because these communities were buffering the need for mental health services. There are community-level programming options that researchers have found increases service utilization among ethnic minorities. For example, researchers have found that the availability of child wellness programs in communities has a positive association with mental health services utilization among ethnic minorities for emerging adults (NeMoyer et al., 2020). More work is needed in this area. As such, there are calls to focus on community and policy factors that are associated with mental health care disparities (Cook et al., 2019).

The higher the percentage of individuals born outside the U.S. in the CSA, the lower the associated coverage rate. This pattern is consistent with previous research indicating that foreign-born individuals in the US have lower mental health service utilization rates (Derr, 2016). This is particularly problematic given the increased stressors they may experience from loss of family networks, previous trauma, discrimination, acculturation pressures, and immigration policies (Rodriguez et al., 2021). Although California’s Medicaid eligibility is extended to foreign-born individuals, there are likely additional barriers and challenges to accessing services. There are structural barriers (e.g., cost, insurance, language), cultural norms and attitudinal preferences (e.g., stigma, group norms), and systemic discrimination that impact service access and participation by the foreign-born community (Derr, 2016). For example, some individuals prefer to seek services from family members, friends, and/or religious leaders rather than formal mental health services (Derr, 2016). Others are more willing to seek services from a medical professional and view the issue somatically rather than emotionally (Derr, 2016). Researchers have found that policies (e.g., Deferred Action for Childhood Arrivals) can have positive impacts on the mental health of foreign-born groups. Further, strengths-based programming that utilizes participatory methods, places foreign-born individuals in decision-making positions, and increasing service access can have positive impacts on subsequent mental wellbeing (Rodriguez et al., 2021).

Lower vehicle ownership within the CSA was associated with higher coverage rates. This result counters the literature that suggests that lack of transportation hampers mental health service access (Cristancho et al., 2016; Kawaii-Bogue et al., 2017; Whetten et al., 2006). However, it may be possible that portions of LA county have robust public transportations systems. Others have found that service areas with stronger public transportation systems have fewer unmet youth mental health needs (Duncan et al., 2020). Our clinic service areas were based on LA data suggesting that individuals travel on average 2 miles to receive mental health services (Maguire-Jack & Klein, 2015). Because the buffer was 2 miles, clinic services may have been close to the youth decreasing the need for transportation. This may reflect more about the proximity of clinics than transportation needs. The size of the service buffer may have also influenced the need for transportation.

College education was the third factor associated with CSA coverage. The higher the percentage of college-educated adults in the CSA the lower the coverage rate. The Prevention and Early Intervention program in our study is intended for individuals with public insurance. Other reports have shown that rates of having a college degree among public insurance beneficiaries is low (Ranji & Salganicoff, 2011). CSAs with higher college attainment would most likely have fewer public insurance beneficiaries which would lead to lower coverage scores.

The more therapists who speak a language other than English in the CSA, the greater the coverage. This finding fits with the literature showing that more bilingual therapists reduces barriers for foreign-born individuals and ethnic minority community members to receive services (Derr, 2016). Agencies involved in the PEI initiative shared that having bilingual therapists was one of their engagement strategies (Regan et al., 2017). Researchers have found that Latino/a therapists involved in PEI made more culturally congruent adaptations to EBPs than White therapists (Ramos et al., 2020). Latino/a therapists frequently made language-based adaptations to the interventions to improve fit with clients. Others have found increased caregiver attendance in PEI youth mental health services when therapists delivered the sessions in languages other than English (Barnett et al., 2020). Bilingual therapists face unique challenges and additional burdens within mental health services systems (Teran et al., 2017). Systems need to provide structural supports to reduce these burdens. Retaining bilingual therapists is important especially given clients’ preference working with bilingual therapists over working through an interpreter (Villalobos et al., 2016).

The higher the proportion of sessions conducted outside the office (e.g., school, home), the higher the coverage rate in the CSA. This pattern fits with the larger mental health service literature. For example, researchers found in a recent meta-analysis that youth utilize mental health services most often in school-based mental health programs, second to outpatient clinics (Duong et al., 2020). However, providing services in locations like schools may have tradeoffs. One PEI study found that caregivers attended far fewer sessions when their youth received services in school (Barnett et al., 2020). Duong and colleagues (2020) found primary care settings were the third most frequent setting for youth with elevated symptoms. The sectors with the lowest provision of mental health services were child welfare and juvenile justice settings. Task-shifting is one approach to increase the reach of mental health services in places like child welfare agencies (Hooley et al., 2021). Providing place-based services – providing care where youth are – is a promising way to increase coverage.

### Implication for Practice and Policy

The findings from this study suggest a few service and policy implications. First, the study demonstrates that meaningful geographic coverage can be calculated with existing administrative claims and census data. Though the literature may not provide clear coverage comparisons, the LACDMH can use the county coverage rate as a benchmark for subsequent planning efforts and goals. For example, the UK monitors their coverage, currently 16% of the targeted adult population, with a goal of 25% (Clark, 2018).

A second service implication is the importance of improving coverage equity. Notwithstanding the explicit focus of PEI serving historically marginalized groups (Los Angeles County Department of Mental Health, 2009), service inequities among ethnic minorities and foreign-born individuals persisted. PEI is intended for public insurance beneficiaries, suggesting that equity in public insurance access could improve equity in PEI coverage. Others have recommended increasing the diversity of the mental health workforce to reduce service access disparities (McGuire & Miranda, 2008). Unfortunately, due to the unreliability of the therapist ethnicity variable in the claims data, it was not possible to assess whether therapist ethnicity influenced coverage. Improving claims data-entry processes to ensure data accuracy would enhance the county’s ability to assess and improve inequities. Monitoring the composition of those who receive services in relation to the composition of the community will allow the LACDMH to adjust when inequities arise.

Next, coverage rates would be much more precise if they were based on client geography. Understandably, client geographic identifiers were not included in the dataset for privacy reasons. To retain client privacy and to improve geographic accuracy, large systems implementing EBPs might consider releasing aggregated geographic data at various geographic levels (e.g., census block group, tract, service planning area). This would preserve client privacy and allow service analysts to examine coverage in more geographically precise ways.

From a systems perspective, the findings of this research have considerable relevance to current and near-term systemic initiatives. First, evidence-based practices were introduced in LA County through the Mental Health Service Act Prevention and Early Intervention initiative but are now provided throughout the outpatient service continuum, with extensive training and support offered for Cognitive Behavioral Therapy and Dialectical Behavior Therapy. In addition, California’s Family First Prevention Services Act (FFPSA) is providing funding for prevention-oriented evidence-based practices to prevent entry into the child welfare system. Los Angeles County’s Department of Child and Family Services has been working with multiple LACDMH, Probation, and other departments to implement these services, which would extend the use of EBPs further. A second area of relevance relates to access to care, both in terms of time and distance that clients must travel to obtain mental health services and the fact that many mental health clinics seek out locations proximal to public transportation. Finally, the finding that greater percentages of bilingual therapists is associated with greater coverage speaks to the absolute need to address the diminishing mental health workforce and significant difficulties recruiting and retaining clinicians. Emerging work across the State focuses on engaging and incentivizing community college students and young adults from diverse cultural backgrounds to pursue careers in public mental health.

### Implications for Scale-up Research

The study also provides implications for mental health services scale-up research. First, small area variation analysis and geospatial methods are underutilized in scale-up mental health research and can yield helpful insights (Townley et al., 2018; Walker et al., 2016), and these methods can be used with existing administrative and census data for both surveillance and research purposes. The present research study highlighted a need for ongoing mental health service surveillance. Other scale-up initiatives have used key performance indicators to assess scale-up success (Grøn et al., 2020), coverage rates could be one indicator. Furthermore, the LACDMH claims system allows the tracking of intervention, service type, service location, provider characteristics, and client characteristics. These sources of data coupled with additional information about the interventions could be used to determine the scalability of future treatments (Milat et al., 2020). These data could be mapped geographically to inform service provision decisions.

Second, the study identifies a set of initial predictors that could inform subsequent scale-up research projects. The constructs from the ExpandNet framework provided a good starting place for predictor selection but lacked enough specificity to operationalize the necessary variables (World Health Organization, 2010). The framework might benefit from separating client-specific determinants from the larger environment construct and identifying client-related characteristics associated with successful scale-up. The empirical literature offered several candidate predictors, but many of them were extrapolated from research on individual-level service utilization. Scale-up researchers in other substantive areas have reported other factors that could be operationalized in subsequent research (Milat et al., 2015). Had this study been testing hypotheses based on this existing literature, minority and impoverished communities would most likely be those with lower coverage rates.

Third, the study underscores the importance of explicitly stating the perspective (e.g., county vs. clinic), the unit of analysis, and the specification of the numerator and denominator when constructing coverage. For example, the coverage rate would have been much lower without the denominator reduction steps. The coverage rate also would have changed if we retained a county perspective rather than a clinic perspective for the CSAs. The clinic perspective counted all the unique clients the clinic served; a county perspective would have only counted unique clients within the county. Making and explicitly reporting decisions about perspective, unit of analysis, and numerator/denominator construction will support better cross-project comparison and better fitting interpretations of the data. There are various ways that scale-up studies have operationalized their numerators and denominators (Charif et al., 2017). The Health Services Coverage framework clearly identified a meaningful outcome variable for this study (i.e., coverage) and provided meaningful guidance for its calculation (Tanahashi, 1978). The present study expands the utility of that framework by suggesting researchers specify what perspective they are using when calculating the coverage rate and offering an example of denominator tailoring steps to approximate the target population more closely.

### Strengths, Limitations, and Future Directions

The present study has several strengths. This is the first study known to us, which calculated a coverage rate for a mental health scale-up initiative in the U.S. outside the Veterans Administration (De Silva et al., 2014). We were able to make these calculations using available census and claims data which exerted minimal burden to LACDMH staff and no additional burden on service providers or clients. In addition, this study created meaningful geography without access to client geographic identifiers using GIS methods. This approach facilitated the construction of predictors fitting with the extant mental health service literature. We were able to explore possible factors associated with coverage using those predictors. In the most recent review on mental health service coverage research, only one study examined predictors of scale-up (De Silva et al., 2014). Notwithstanding these strengths, the study also has several limitations.

The findings from this study should be viewed within the context of its constraints and limitations. First, our denominator specification process included mental illness prevalence rates that did not account for insurance type. PEI is intended for youth with public health insurance, and youth with public insurance have higher prevalence rates of mental illness (Ghandour et al., 2019). Available youth mental illness prevalence data that include insurance type lacks diagnostic specificity and condition severity, which leads to lower prevalence rates in those data compared to the prevalence rates we used. Our prevalence rates closely mirror Medicaid prevalence rates for adults (Adelmann, 2003), but there still is the possibility that we have underestimated the number of PEI qualifying youth. An important area for future study is to gather mental health epidemiologic data for publicly insured youth.

Second, this study uses administrative claims data to index EBP coverage. Claims reflect therapists’ self-report of the delivery of EBPs to individual clients as covered in their contracts with LACDMH. Although there are EBP specific implementation guidelines related to initial and ongoing training requirements for therapists to claim for an EBP, adherence to the EBP is not measured at the system level and it is not known whether a full course of treatment was delivered.

Third, our findings use census and administrative data from Los Angeles County for a youth-specific program which limits generalizability. Our findings are specific to Los Angeles County with its unique geography, population composition, and county incentive structure for EBP use. Our findings are also specific to youth mental health services. The role that caretakers and families play on youth accessing services is distinct compared to adults accessing care. Administrative claims data and census data are useful and practical and they have limits given they are not designed for research. There are a few different sources of population-level data, we selected those sources which provided rigorous and fitting estimates for the phenomena under study.

Fourth, the observational nature of the data, the sample size, and narrow availability of variables limited our ability to use other robust causal approachs (e.g., instrumental variables) to better understand the inter-relationships among the predictors. Various variables in the model were correlated. For example, communities with greater poverty had fewer vehicles. Communities with higher population density also had fewer vehicles. We made corrections to the final analytic model to reduce the influence of autocorrelation and heteroskedasticity. We retained the variables in the model given their empirical/theoretical support from the literature, their relevance to service system administrators and policy makers, and their individual variance inflation factor scores.

Finally, this study used a GIS approach to approximate the coverage of a mental health service initiative. The coverage rates would have been more precise using client geographic identifiers, like zip code, but they were not available due to privacy concerns. Notwithstanding this constraint, we used geographic data from LA county-based studies to create meaningful clinic catchment areas (Guerrero et al., 2013; Guerrero & Kao, 2013; Maguire-Jack & Klein, 2015). Retaining the 2-mile buffer coincided with previous research, approximated the average distance between a large sample of the clinics, and prevented total geographic overlap for clinics in highly dense areas. Even with the statistical corrections applied using a robust variance estimator, some influential observations and issues with linearity remained, and we should assume that a degree of spatial auto-correlation persisted. Hence, our results should be interpreted in light of these limitations.

This study prompted several areas of future research. Subsequent research could add land-use characteristics as a layer in the GIS data to account for where people within the geographic unit live. While beyond the scope of the current paper, researchers could explore the possibility of using spatial autoregressive models with coverage rate data. Third, if available, researchers could include data that would account for differences in clinic service capacity. And finally, our findings are specific to LA County. Replicating this research in other geographic locations could help to determine the generalizability of our findings.

## Conclusion

This study ascertained the coverage of multiple EBPs delivered within a system-driven implementation of EBPs for youth in public mental health services in LA County. Overall, the coverage rate of EBPs selected for implementation is 17% of the target population. This rate tracks with other large-scale implementation efforts (Clark, 2018) and provides an initial benchmark for subsequent efforts to improve the coverage of evidence-based mental health services for children and youth. Within a large, diverse county, there were regional differences in coverage rates. Neighborhood-level factors such as the proportion of ethnic minorities, foreign-born individuals, and individuals with a college degree were negatively associated with coverage rates within clinic service areas. These findings provide target communities for outreach to facilitate improved access. This study represents one of the first to examine factors associated with the scale-up of evidence-based mental health care and offers methods to calculate meaningful coverage rates and predictors based on administrative and publicly available data.

## Electronic Supplementary Material

Below is the link to the electronic supplementary material.

Supplementary Material 1

## Data Availability

Requests for data may be sent to the corresponding author.

## List of Abbreviations

EBP: Evidence-based practices
LACDMH: Los Angeles County Department of Mental Health
PEI: Prevention and Early Intervention initiative

## Declarations

### Reporting Guideline

We followed the geospatial components of the Spatial Lifecourse Epidemiology Reporting Standards (ISLE-ReST).
