# Correction to: Scaling-up Child and Youth Mental Health Services: Assessing Coverage of a County-Wide Prevention and Early Intervention Initiative During One Fiscal Year

**DOI:** 10.1007/s10488-023-01258-x

**Published:** 2023-03-01

**Authors:** Cole Hooley, Deborah Salvo, Derek S. Brown, Lauren Brookman-Frazee, Anna S. Lau, Ross C. Brownson, Patrick J. Fowler, Debbie Innes-Gomberg, Enola K. Proctor

**Affiliations:** 1 Brigham Young University, 84602 Provo, UT USA; 2 Brown School, Washington University in St. Louis, 1 Brookings Drive, 63130 St. Louis, MO USA; 3 Department of Psychiatry, University of California San Diego, 9500 Gilman Drive #0812, 92093 La Jolla, CA USA; 4 Department of Psychology, UCLA, 502 Portola Plaza, 90095 Los Angeles, CA USA; 5 Prevention Research Center, Brown School, Department of Surgery, Division of Public Health Sciences, and Alvin J. Siteman Cancer Center, Washington University in St. Louis, Washington University School of Medicine, Washington, University in St. Louis CDC U48DP006395, the Foundation, for Barnes-Jewish Hospital, 1 Brookings Drive, 63130 St. Louis, MO USA; 6 Los Angeles County Department of Mental Health, 510 S., Vermont Avenue, 17Th Floor, 90020 Los Angeles, CA USA; 7 Department of Kinesiology and Health Education, The University of Texas at Austin, Bellmont Hall 822J, 2109 San Jacinto Blvd, Stp D3700, 78712 Austin, TX USA

**Correction to: Administration and Policy in Mental Health and Mental Health Services Research (2023) 50:17–32** 10.1007/s10488-022-01220-3

The article “ Scaling-up Child and Youth Mental Health Services: Assessing Coverage of a County-Wide Prevention and Early Intervention Initiative During One Fiscal Year”, written by Cole Hooley, Deborah Salvo. Derek S, Brown, Lauren Brookman-Frazee, Anna S. Lau,Ross C. Brownson, Patrick J. Fowler. Debbie Innes-Gomberg, Enola K. Proctor was originally published Online First without Open Access. After publication in volume 50, issue 1, page 17–32 the author decided to opt for Open Choice and to make the article an Open Access publication. Therefore, the copyright of the article has been changed to © The Author(s) 2023 and the article is forthwith distributed under the terms of the Creative Commons Attribution 4.0 International License, which permits use, sharing, adaptation, distribution and reproduction in any medium or format, as long as you give appropriate credit to the original author(s) and the 0source, provide a link to the Creative Commons licence, and indicate if changes were made. The images or other third party material in this article are included in the article’s Creative Commons licence, unless indicated otherwise in a credit line to the material. If material is not included in the article’s Creative Commons licence and your intended use is not permitted by statutory regulation or exceeds the permitted use, you will need to obtain permission directly from the copyright holder. To view a copy of this licence, visit http://creativecommons.org/licenses/by/4.0.

