# Supplementary material for: Scaling-up Child and Youth Mental Health Services: Assessing Coverage of a County-Wide Prevention and Early Intervention Initiative During One Fiscal Year
Source: Adm Policy Ment Health. 2022 Oct 26;50(1):17–32. doi: 10.1007/s10488-022-01220-3 (PMC9977707; doi:10.1007/s10488-022-01220-3)
Supplement: Supplementary file 1 — Supplementary Material 1 [file 10488_2022_1220_MOESM1_ESM.docx]

**Supplementary Materials**

# **Table S1.** Description of geospatial shapefiles used in this study.

| **File name** | **Type** | **Source** | **Description** |
| --- | --- | --- | --- |
| DRP_COUNTY_BOUNDARY.shp | Polygon | <https://egis3.lacounty.gov/dataportal/drp_county_boundary/> | LA County boundary file including Catalina & San Clemente islands. From LA county GIS portal. Pulled in 2019 |
| tl_2014_06_bg.shp | Polygon | <https://www.census.gov/cgi-bin/geo/shapefiles/index.php?year=2014&layergroup=Block+Groups> | California census block groups TIGER shapefile for 2014 |
| Service_Planning_Areas_2012.shp | Polygon | <https://egis3.lacounty.gov/dataportal/2012/03/01/service-planning-areas-spa-2012/> | Service planning areas for LA county. These areas are used by the department of mental health. These are aggregated from 2010 census tracts and the file was created in 2012. |
| tl_2014_06037_roads.shp | Line | <https://www.census.gov/cgi-bin/geo/shapefiles/index.php?year=2014&layergroup=Roads> | All roads LA county from 2014 TIGER/Line shapefile |
| Street_Addresses_US.lox | Point | ESRI | ESRI ArcMap 2012 address database to match clinic addresses |
| DISS-lacmhd-program-addresses.csv | Point | LACDMH claims data | LA County Department of Mental Health agencies who provided PEI services during fiscal year 2013-2014 |

# **Table S2.** Unit of analysis and numerator/denominator calculations for each research question.

| **Research question** | **Unit of analysis (n)** | **Coverage numerator** | **Coverage denominator** |
| --- | --- | --- | --- |
| #1 – PEI coverage | County (n=1) | Distinct clients who received at least one psychotherapy session within the county | PEI target population within county |
| #2 - Factors associated with coverage | Clinic Service Areas (CSA) (n=254) | Distinct clients who received at least one session from the clinic. | PEI target population within CSA |

# **Table S3.** Contact coverage denominator reduction steps using zip code 90011 as an example

| **Step** | **Description** |  |  |  |  |  |
| --- | --- | --- | --- | --- | --- | --- |
| 1 | Initial denominator (youth population) | 35,247 |  |  |  |  |
| 2 | Medi-Cal eligible | 35,247 | x | 45.3% | = | 15,967 |
| 3 | Any mental disorder prevalence | 15,967 | x | 32.8% | = | 5,237 |
| 4 | Non-serious disorders | 5,237 | x | 92% | = | 4,818 |
| 5 | Likely to seek services | 4,818 | x | 50.6% | = | 2,438 |
|  | Specified denominator | 2,438 |  |  |  |  |


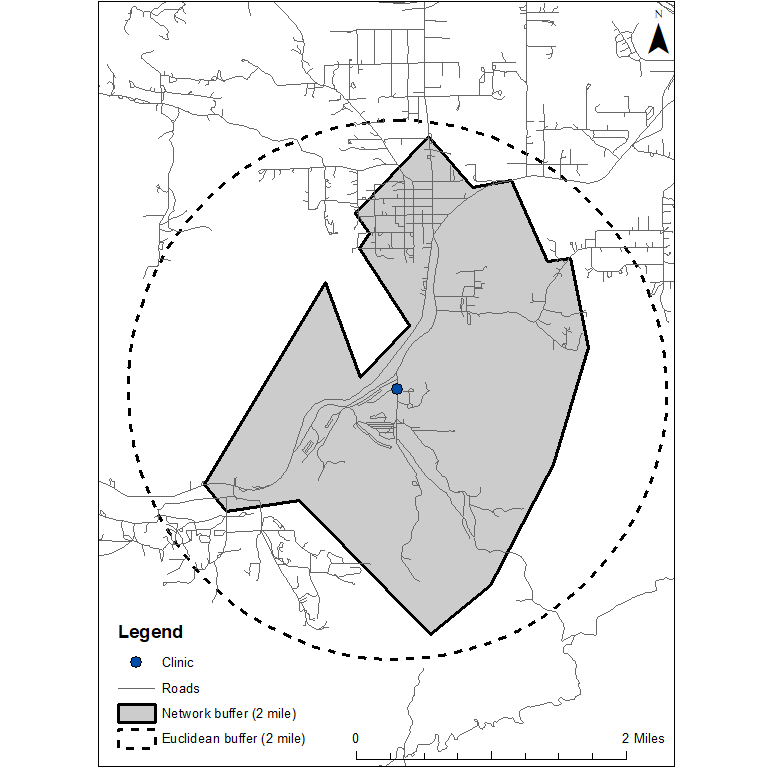


# ***Figure S1.*** *An example of the clinic service area buffer used for this study, compared to a Euclidian buffer.*
